# Glucagon-Like Peptide-1 Receptor Agonists for Arthritis and Osteoarthritis

**DOI:** 10.7759/cureus.93373

**Published:** 2025-09-27

**Authors:** Abdulqadir J Nashwan

**Affiliations:** 1 Nursing and Midwifery Research, Hamad Medical Corporation, Doha, QAT

**Keywords:** arthritis, glucagon-like peptide-1 receptor agonists, gout, inflammation, osteoarthritis, rheumatoid arthritis

## Abstract

Glucagon-like peptide-1 receptor agonists (GLP-1RAs) play a pivotal role in managing type 2 diabetes mellitus (T2DM), obesity, and other conditions, with proven benefits including, but not limited to, weight loss, glycemic control, and cardiovascular protection. However, their implications for arthritis are still debatable. GLP-1RAs may reduce systemic inflammation, alleviate joint stress through overall weight loss, and exhibit chondroprotective effects. Nevertheless, concerns such as augmented gout flares, joint pain, and indefinite long-term outcomes temper their promise. This editorial highlights the dual nature of GLP-1RAs in arthritis, presenting them as both potential benefits and risks, contingent upon the specific arthritis subtype and individual patient profiles.

## Editorial

Glucagon-like peptide-1 receptor agonists (GLP-1RAs) have recently gained attention as novel agents, primarily in the management of type 2 diabetes mellitus (T2DM) and obesity (two conditions that disproportionately affect older adults) [1]. Their ability to promote weight loss, regulate blood glucose, and provide cardioprotective effects has positioned them at the forefront of therapeutic innovation, especially for aging populations [1]. However, their interaction with arthritis, encompassing a range of joint disorders, including rheumatoid arthritis (RA), osteoarthritis (OA), and gout, has sparked scientific controversy.

This editorial synthesizes randomized controlled trials, observational studies, and key preclinical reports published between 2018 and 2025 to explore the potential benefits and risks of GLP-1RAs in the context of OA, RA, and gout. Rather than providing definitive answers, the goal is to highlight areas of convergence and controversy, stimulate critical discussion, and outline priorities for future research.

GLP-1RAs mimic the GLP-1 incretin hormone, which is secreted in response to nutrient intake [2]. GLP-1RAs improve the secretion of glucose-dependent insulin, slow gastric emptying, and suppress glucagon release [2]. Notably, these receptors are expressed in the pancreas and other vital organs, including the brain, heart, and immune cells, suggesting that these agents have a wide range of systemic effects [3].

Emerging evidence suggests that GLP-1RAs exert anti-inflammatory effects, which may benefit individuals with arthritis [4]. For instance, Lee et al. (2022) demonstrated that GLP-1RAs consistently reduced inflammatory markers (e.g., interleukin-6 (IL-6) and C-reactive protein (CRP)) in patients with T2DM [5]. These cytokines are also implicated in the pathogenesis of RA and OA, suggesting potential cross-disease benefits [5]. However, these findings remain hypothesis-generating as they are largely limited to biomarker-level data rather than patient-level clinical endpoints. To date, no randomized controlled trials have specifically evaluated GLP-1RAs for arthritis outcomes such as pain reduction, functional improvement, or disease activity scores in RA or OA. Dedicated trials with disease-specific endpoints are required to determine whether these biomarker effects translate into meaningful clinical benefit.

Obesity is a major risk factor for OA, as excessive weight exacerbates joint mechanical stress. GLP-1RAs, particularly semaglutide, have been shown to induce substantial weight loss [6]. A trial by Wilding et al. (2021) reported an average weight reduction of 15% among participants, which may potentially alleviate joint stress and delay the progression of OA [7]. However, no data from this or other GLP-1RA trials have evaluated structural modification endpoints (e.g., radiographic joint space narrowing or magnetic resonance imaging (MRI) cartilage thickness). Therefore, while weight loss may plausibly delay OA progression, evidence for disease-modifying effects remains lacking, and current benefits should be interpreted as symptomatic and functional improvements rather than proven structural protection.

Recent findings from a 2024 randomized controlled trial further support these benefits among patients with obesity and knee osteoarthritis; once-weekly semaglutide led to significantly greater weight loss and more substantial reductions in knee pain and stiffness compared to placebo (n=407; follow-up 68 weeks; between-group difference in Western Ontario and McMaster Universities Osteoarthritis Index (WOMAC) pain = -14.2 (95% CI, -19.3 to -9.1); p<0.001), with gastrointestinal events being the most common reason for discontinuation [8]. These improvements were accompanied by enhanced physical function, reinforcing the potential of semaglutide as a dual-action therapy for managing weight and OA symptoms [8]. Complementing this, a 2025 network meta-analysis by Jiang et al. compared various antidiabetic agents in obese patients with knee OA and found that while metformin ranked highest in pain relief, semaglutide showed a balanced efficacy and safety profile with a low risk of serious adverse events [9].

Additionally, in the Shanghai Osteoarthritis Cohort, GLP-1RA therapy in patients with knee OA with comorbid T2DM was associated with greater weight loss (−7.29 kg; p<0.001), lower knee surgery incidence (1.7% vs 5.9%; p=0.014), and significant improvements in WOMAC total (−1.46; p=0.038) and pain subscores (−3.37; p=0.007) compared with non-users. Cartilage-loss velocity of the medial femorotibial joint was significantly slower (−0.02 mm; p=0.004), and within-group analyses showed reduced analgesic use and cartilage loss after GLP-1RA initiation [10]. In contrast, Wei et al. (2024) evaluated all-cause mortality risk in patients with knee or hip OA treated with anti-obesity medications, but did not specifically stratify by rate of weight loss. Evidence remains limited regarding whether the speed of weight loss, rather than the absolute amount, affects long-term mortality or joint outcomes [11-12]. Further research is needed to disentangle the effects of weight-loss rate versus total weight loss in this population.

On the other hand, preclinical studies have shown the direct protective effects of GLP-1RAs on cartilage. For instance, animal models have found that liraglutide reduces cartilage degradation and synovial inflammation in OA models, likely through the modulation of oxidative stress and inflammatory pathways [13]. However, these findings face major translational limitations, including differences in dosing and pharmacokinetics between animals and humans, the relevance of surgically induced OA models to human disease, and the short experimental timelines that may not capture long-term disease progression. To date, no human trials have demonstrated structural disease modification based on these mechanisms. Specific translational studies are needed before advancing to outcomes trials, including phase II studies that incorporate biomarkers of cartilage turnover (e.g., C-terminal telopeptide of type II collagen (CTX-II) and Cartilage Oligomeric Matrix Protein (COMP)) and imaging endpoints such as MRI-based cartilage thickness.

Additionally, GLP-1RAs offer systemic benefits that may enhance overall joint health [14]. The research team analyzed data from the Department of Veterans Affairs (USA) to construct a large cohort of 215,970 patients with diabetes who initiated GLP-1RA therapy and compared them with over 500,000 matched individuals initiating other antihyperglycemic agents or continuing usual care [14]. Using a discovery approach across 175 prespecified health outcomes, the investigators found that GLP-1RA use was associated with lower risk of several inflammatory and cardiometabolic conditions-for example, reduced risk of major adverse cardiovascular events (HR 0.82, 95% CI 0.78-0.87) and Alzheimer’s disease (HR 0.86, 95% CI 0.80-0.92)-but higher risk of gastrointestinal disorders and arthritic events, including gout flares (HR 1.22, 95% CI 1.11-1.33), new osteoarthritis diagnoses (HR 1.15, 95% CI 1.08-1.22), and rheumatoid arthritis exacerbations (HR 1.18, 95% CI 1.05-1.32).

These associations must be interpreted cautiously, as the study was observational in nature and subject to residual confounding despite extensive adjustment. The results nevertheless provide important insights into the benefits and risks of GLP-1RAs and may be useful for informing clinical care and guiding research agendas [14]. However, due to the observational nature of the analysis, despite extensive covariate adjustment, residual confounding and indication bias cannot be excluded, and these findings should be carefully interpreted.

Although the exact mechanisms are not fully elucidated, these findings highlight the importance of proactive musculoskeletal monitoring during GLP-1RA therapy. Monitoring should follow a structured approach: (1) obtain a baseline serum urate level in all patients with a history of gout; (2) consider short-term prophylactic colchicine (e.g., 3-6 weeks) in those with serum urate above 6.8 mg/dL or with prior frequent flares; (3) reassess serum urate and clinical symptoms within 1-3 months of initiating GLP-1RA therapy; (4) monitor periodically for new or worsening joint pain, stiffness, or arthritic symptoms; (5) perform baseline bone mineral density assessment only if risk factors for osteoporosis are present; and (6) refer high-risk patients with recurrent gout flares to rheumatology for prophylaxis and long-term management, in accordance with current rheumatology guidance (e.g., 2020 American College of Rheumatology Guideline for the Management of Gout).

For patients at elevated risk of osteoporosis or fracture, a baseline bone mineral density (BMD) measurement can be considered in accordance with existing osteoporosis guidelines. Incorporating these measures into routine follow-up can help detect musculoskeletal complications early and guide individualized management [15].

Recently, concerns have been raised about the potential adverse effects of GLP-1RAs in specific arthritis subtypes despite their promising benefits. Moreover, the rapid weight loss induced by GLP-1RAs can increase the risk of gout flares due to the mobilization of urate stores. A higher incidence of gout attacks was observed among GLP-1RA users within the first 6 months of treatment [16]. Also, some patients on GLP-1RAs report musculoskeletal side effects, including joint pain [16]. Although GLP-1RAs have been in clinical use for metabolic indications for several years, long-term musculoskeletal outcomes-particularly disease-specific, structural endpoints in OA and RA-remain underexplored [17-18]. For example, while weight loss may relieve mechanical joint stress, its effects on bone density and cartilage turnover warrant further investigations.

To sum up, GLP-1RAs may influence arthritis outcomes in both beneficial and adverse ways. Their weight-loss and anti-inflammatory effects can improve pain and function in osteoarthritis and possibly other musculoskeletal conditions, while early gout flares and musculoskeletal adverse events remain important risks. Current evidence is insufficient to recommend GLP-1RAs as disease-modifying or primary treatments for RA, OA, or gout, and their use should remain guided by metabolic indications (T2DM, obesity) with individualized risk-benefit discussions.

Future research should prioritize randomized controlled trials and pragmatic comparative effectiveness studies in patients with OA, RA, and gout, stratified by baseline urate levels, inflammatory burden, and comorbid metabolic disease. These trials should include patient-centered endpoints (pain, function, flare frequency), structural outcomes (joint space narrowing, MRI cartilage thickness), and safety surveillance for early gout flares and musculoskeletal events. Longitudinal registry studies and pharmacoepidemiologic analyses can complement trial data to characterize rare adverse events and subgroup effects.
